# Management of Tenosynovial Giant Cell Tumor: A Neoplastic and Inflammatory Disease

**DOI:** 10.5435/JAAOSGlobal-D-20-00028

**Published:** 2020-11-02

**Authors:** John H. Healey, Nicholas M. Bernthal, Michiel van de Sande

**Affiliations:** From the Department of Surgery, Orthopaedic Service, Memorial Sloan Kettering Cancer Center, New York, NY (Healey); Department of Orthopaedic Surgery, David Geffen School of Medicine at UCLA, Santa Monica, CA (Bernthal); and Department of Orthopedics, Leiden University Medical Center, Leiden, Netherlands (van de Sande).

## Abstract

**Background:**

Patients with diffuse tenosynovial giant cell tumor (TGCT) face a high risk of recurrence, progression, and disability. This systematic review assesses the recent evidence of surgical, adjuvant, and systemic treatments for TGCT.

**Methods:**

We searched PubMed and Ovid with the terms “Giant cell tumor of tendon sheath” OR “pigmented villonodular synovitis” OR “tenosynovial giant cell” AND “treatment” OR “surgery.” Inclusion criteria: published 2013 to present; prospective or retrospective design; English language; > 20 patients with histopathological confirmed diagnosis of TGCT; and ≥ 1 efficacy and/or safety outcome from surgery, systemic drug therapy, or adjuvant ^90^yttrium radiosynoviorthesis.

**Results:**

Of the 434 studies identified, 25 met the inclusion criteria. Of 11 studies in patients with disease in the knee, nine examined surgical treatment approaches, and two evaluated adjuvant ^90^yttrium radiosynoviorthesis. Of 11 studies in patients with mixed sites of disease, six assessed surgical treatment approaches, and five evaluated systemic drug therapies. Three studies assessed surgery in patients with TGCT in the hand, hip, and ankle or foot.

**Discussion:**

The high rates of recurrence and risks associated with surgery emphasize the need for novel treatments in patients with symptomatic, advanced TGCT. Systemic therapy may be valuable as part of a multidisciplinary approach.

Tenosynovial giant cell tumors (TGCTs) are rare, locally aggressive, typically benign neoplasms of joints, bursae, and tendon sheaths.^1-3^ Symptoms of TGCT include pain, stiffness, swelling, and limitation in range of motion. TGCTs have a wide clinical spectrum and affect patients of all ages. Until the World Health Organization reclassified them in 2013,^1,2^ TGCTs were classified according to their site of origin (ie, bone, soft tissue, synovium, or tendon sheath) as giant cell tumor of tendon sheath (GCTTS) or nodular tenosynovitis and diffuse type giant cell tumors or pigmented villonodular synovitis (PVNS).^4^ According to the 2013 World Health Organization reclassification, the term localized TGCT encompasses GCTTS and nodular tenosynovitis, whereas diffuse TGCT encompasses diffuse-type giant cell tumor and PVNS.^1,2^ This reclassification emphasizes that the driving force in the pathology of TGCT is a tumor and that the symptoms are often defined by a secondary inflammatory joint response.

Compared with localized TGCT, diffuse TGCT tends to affect a younger population and is more predominant in women than men.^2^ Development of TGCT has been associated with a clonal neoplastic process, often involving a specific chromosomal translocation, t(1;2) (CSF-1;COL6A3), resulting in the overexpression of colony-stimulating factor 1 and recruitment of CSF1 receptor (CSF1R) macrophages, giant cells, and osteoclasts.^3^ A subset of tumors exists (2% to 16%) where there seems to be another effector downstream that results in excess CSF1R expression/response without the mutation.^5,6^

Updated data on TGCT epidemiology exist. A 2017 Danish registry analysis reported a population prevalence of 44 per 100,000 for localized TGCT and 12 per 100,000 for diffuse TGCT, and a 10-year risk of recurrence of 9.8% for localized TGCT and 19.1% for diffuse TGCT.^7^ A 2017 Dutch registry study reported standardized worldwide incidence rates of 29 cases per million for TGCT affecting digits, 10 for localized TGCT, and four for diffuse TGCT. In the study, the recurrence rate was 2.6 times higher for diffuse TGCT compared with localized TGCT.^8^

There is currently no consensus on the optimal standard of care for TGCT, especially for patients with widely diffuse or recurrent disease. Three European groups have previously published recommendations.^3,4,9^ In 2012, a combined UK and Dutch group proposed an integrated, multidisciplinary treatment protocol for TGCT.^4^ A 2016 UK guideline stated that patients with TGCT are usually treated by surgery alone and that, rarely, radiotherapy or imatinib may be used.^9^ In addition, in 2016, an Italian group remarked on the lack of high-quality studies of TGCT treatment and identified open surgical excision as the benchmark treatment of diffuse disease.^3^ Most patients with diffuse TGCT are treated by arthroscopic or open surgical excision with partial or extensive synovectomy.^10^ The most appropriate form of surgery has not been identified and probably differs by tumor site and location.^11^

Radiosynoviorthesis (RSO) and external beam radiotherapy (EBRT) are sometimes used alone or in combination with surgery. Their contribution is unclear because of limited low-level of evidence (LOE), poor-quality data, and contradictory results. The authors of a 2015 meta-analysis of individual patient data from 35 observational studies reported what they deemed to be very low-quality evidence that perioperative EBRT might reduce the rate of recurrence in patients with diffuse PVNS.^12^ The main issues with RSO are morbidity to the joint, including early onset arthritis, osteonecrosis, increased postradiotherapy perioperative risks for complications, and concerns with malignant transformation or secondary radiation-associated sarcomas.^12^

In managing patients with advanced TGCT, surgeons must balance the potential benefits and harms of different surgical and nonsurgical approaches with the risk of recurrence, disease progression, and long-term disability. Given the paucity of proven, effective local or systemic therapies, clinicians have struggled in this regard to manage refractory disease. Notable advances in treatment options for diffuse TGCT have recently been made, which suggest a potential role for systemic therapies as part of the overall management of the disease process. This systematic review assesses the recent evidence of treatment strategies for patients with TGCT, providing a critical, up-to-date assessment of trends while highlighting continued unmet needs.

## Methods

### Search Strategy

On January 30, 2020, we searched MEDLINE (via PubMed) and EMBASE using the following search terms: ([GCTTS] OR [PVNS] OR [tenosynovial giant cell]) AND (treatment OR surgery). We set the publication date search limits from January 1, 2013, to January 31, 2020, for PubMed and 2013 to 2020 for EMBASE. We limited the searches to English-language publications. The EMBASE searches excluded conference abstracts/articles, case reports, preclinical studies, reviews, editorials, letters, notes, chapters, and surveys.

### Eligibility Criteria

We included prospective or retrospective studies that reported > 1 efficacy and/or safety outcome from surgery, systemic drug therapy, or adjuvant ^90^yttrium RSO in > 20 patients with a histopathologically confirmed diagnosis of TGCT, GCTTS, or PVNS. Reasons for study exclusion were having ≤ 20 patients with TGCT, EBRT as the primary treatment modality, lack of confirmed histopathologic diagnosis, mixed population with no separate reporting of data for patients with TGCT, patient-reported outcomes as the only efficacy measures, and lack of an abstract.

### Study Selection

After removal of duplicates from PubMed and Ovid searches, the title and abstract were screened to exclude records not meeting all the inclusion criteria. Studies meeting the initial screening underwent repeat screening through full-text review to verify all the information necessary for complete application of inclusion criteria.

### Data Extraction and Analysis

From the included studies, we extracted and recorded the following data: publication year, study design, treatment period, number of patients, type and anatomic site of TGCT, type of treatment of TGCT, length of follow-up, key efficacy end points, and main complications or adverse events (AEs). Wide heterogeneity in study design, end points, and types of treatment prevented quantitative statistical analyses. We applied the criteria outlined in the *J Am Acad Orthop Surg* author instructions to assign an LOE for each included study.

## Results

**Figure 1:**
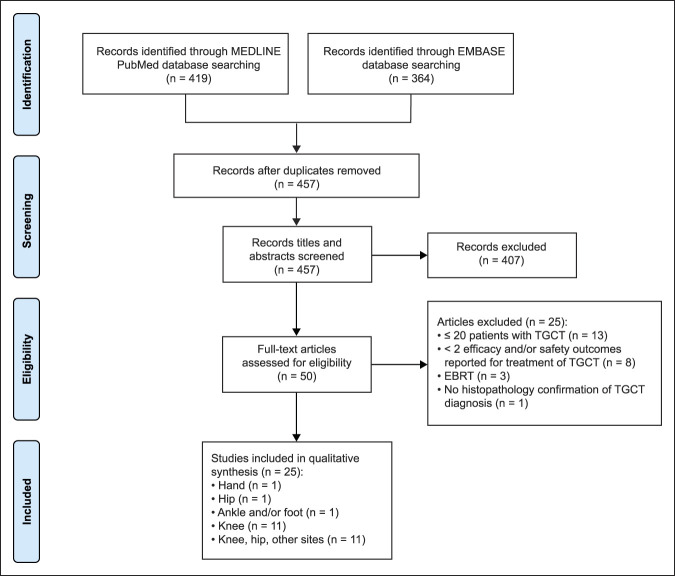
Chart showing the PRISMA flow diagram. EBRT = external beam radiotherapy, TGCT = diffuse tenosynovial giant cell tumor

As illustrated in the PRISMA^13^ chart in Figure 1, of 457 studies identified on screening, 25 studies met all the inclusion criteria. Of 11 studies in patients with disease in the knee, nine studies examined surgical treatments^14,15,16,17,18,19,20,21,22^ and two studies evaluated adjuvant ^90^yttrium RSO^23,24^ (Table 1). Among the 11 studies in patients with heterogeneous sites of disease, six reported the results of surgical treatment^11,25,26,27,28,29^ and five evaluated systemic drug therapies^30,31,32,33,34^ (Table 2). Three studies (1 study each) reported the results of surgical outcomes in patients with TGCT in the hand,^35^ hip,^36^ and foot or ankle^37^ (Table 3). Except for four systemic drug therapy studies with prospective clinical trial design, all other studies had a retrospective cohort design (LOE III). The prospective studies included 2 phase 1 trials (LOE II),^30,32^ 1 phase 2 trial (LOE II),^31^ and 1 randomized controlled trial (RCT; LOE I).^33^

**Table 1:** Summary of Studies in Patients With TGCT in Knee

Study (No. of pts)	Study Design (Tx Period), LOE	Dx, F/U	Tx	Efficacy^a^	Complications^a^
**Surgery**					
Colman et al^14^ (n = 48)	Retrospective cohort (1993-2011) LOE: III	Diffuse PVNS F/U: 40^b^ mo	All arthroscopic Sv (n = 26) versus open/arthroscopic Sv (n = 11) versus open/open Sv (n = 11)	Recurrence: total (50%); all arthroscopic (62%); open/arthroscopic (9%); open/open (64%) (*P* = 0.008) Multiple recurrences: total (27%); all arthroscopic (35%); open/arthroscopic (0%); open/open (36%) (*P* = 0.07)	All arthroscopic: hemarthrosis (8%); DVT (4%) Open/arthroscopic: hemarthrosis (9%); stiffness requiring MUA (9%) Open/open: superficial wound infection (9%)
Georgiannos et al^15^ (n = 44)	Retrospective cohort (1990-2006) LOE: III	Localized PVNS F/U: 12^c^ yr	Arthroscopically assisted mini-open partial Sv (n = 21) versus arthroscopic excision of lesion (n = 23)	From preop to last follow-up, mean Lysholm and Ogilvie-Harris scores improved in both groups (*P* < 0.05); no differences between groups (*P =* 0.06) Recurrence: mini-open partial Sv (4.8%); arthroscopic excision of lesion (0) (*P* = 0.1)	CRPS: mini-open partial Sv (10%); arthroscopic excision of lesion (0) (*P =* 0.06) No other complications
Gu et al^16^ (n = 41)	Retrospective cohort (2002-2010) LOE: III	Diffuse PVNS F/U: 2.9^c^ yr	Anteroposterior open surgical resection (n = 20) versus modified multidirectional arthroscopic surgery (n = 21)	IKDC and Lysholm scores were similar between groups preop and higher in arthroscopic surgery group at 1-yr and 3-yr postop (*P* < 0.001) Recurrence: open surgery (22%); arthroscopic surgery (6%) (*P* ≥ 0.05)	Postop bleeding (mL): open surgery (332 ± 79); arthroscopic surgery (154 ± 44) (*P* < 0.001) Complications: NR
Jabalameli et al^17^ (n = 26)	Retrospective cohort (1996-2012) LOE: III	Localized PVNS (n = 11) or diffuse PVNS (n = 15) F/U: 4.6^c^ yr	Subtotal Sv (n = 5) or total Sv (n = 21)	Recurrence: 7.7% Mean KSS: preop (63.1 ± 6.7); postop (77.8 ± 9.3) (*P* < 0.009)	No postop knee instability, infection, or neurovascular injury
Jain et al^18^ (n = 40)	Retrospective cohort (1987-2012) LOE: III	Localized PVNS (n = 11) or diffuse PVNS (n = 29) F/U: 7^c^ yr	Local excision for localized PVNS; arthroscopic Sv for diffuse PVNS	Recurrence: first (30%; 3-12 mo postop); second (12%; 14-18 mo postop); third (5%; 2-yr postop) RFS: 1-yr (69%); 2-yr (82%); 5-yr (57%)	No postop infection, neurovascular damage, DVT, or wound healing
Keyhani et al^19^ (n = 21)	Retrospective cohort (2009-2012) LOE: III	Diffuse PVNS F/U: 5^c^ yr	Arthroscopic complete Sv	Local recurrence: 10% (without clinical signs during follow-up) ROM:^c^ preop (83 ± 6), postop (127 ± 7) (*P* < 0.001) Lysholm score:^c^ preop (49 ± 2), postop (81 ± 4) (*P* < 0.001) IKDC score:^c^ preop (47 ± 1), postop (79 ± 1) (*P* < 0.001)	No op or postop complications (ie, no swelling, infection, joint stiffness, or neurovascular lesions)
Patel et al^20^ (n = 214)	Retrospective cohort (2002-2015) LOE: III	Localized PVNS (n = 100) or diffuse PVNS (n = 114) F/U: 25^c^ mo	Surgery (n = 184 pts; 86%): arthroscopic Sv (n = 23); combined arthroscopic and open Sv (n = 4); open anterior or posterior Sv (n = 46); open anterior and posterior Sv (n = 42); open localized Sv (n = 65); distal femur arthroplasty (n = 1); TKA (n = 3) Conservative management (n = 28; 13%): 14% had surgery at later stage	Successful tx (no need for further surgery): 87.5% Recurrence: first (30%); second (6.5%) Arthroscopic versus open recurrence: localized PVNS (9.1% versus 8.6%; *P* > 0.05); diffuse PVNS (83.3% versus 44.8%; RR = 1.86; 95% CI, 1.32–2.62; *P* = 0.0004)	Postop: 9.8% (of which 89% were from open surgery) Types of postop complications: Superficial wound infection (n = 6); foot drop (n = 3); hemarthrosis (n = 3); stiffness requiring manipulation under anesthesia (n = 2); complex regional pain syndrome (n = 2); blistering from tourniquet (n = 1); DVT (n = 1)
van der Heijden et al^21^ (n = 30)	Retrospective cohort (1980-2011) LOE: III	DTGCT F/U: 64^c^ mo	Open Sv (n = 14) or arthroscopic Sv (n = 16)	Recurrence: initial open Sv (n = 4; 29%); initial arthroscopic Sv (n = 15; 94%) Of pts tx'd with initial arthroscopic Sv, 15 underwent open Sv	Initial open Sv: recurrent hemarthrosis and secondary OA (n = 1); multiple recurrences and OA of knee after 3 open Sv (n = 1) Initial arthroscopic Sv: recurrent disease and OA (n = 2)
Verspoor et al^22^ (n = 91; study also included some demographic data on 13 with PVNS in other areas)	Retrospective cohort (1985–2011) LOE: III	Primary and recurrent, localized PVNS (n = 27) or diffuse PVNS (n = 64) F/U: 7.0^c^ yr	Localized PVNS: All pts treated by open or arthroscopic Sv Diffuse PVNS: Open or arthroscopic synovectomy (n = 53);^90^yttrium RSO (n = 7); unknown (n = 4)	Localized PVNS: RFS: 1-yr (83%); 5-yr (69%) Diffuse PVNS: RFS: 1-yr (68%); 5-yr (32%); 10-yr (25%); 15-yr (16%)	Localized PVNS: Postop: superficial wound infection (n = 1); deep infection (n = 1); femoral nerve neuropathy (n = 1) Diffuse PVNS: Postop: delayed wound healing (n = 2); local paresthesia (n = 1); stiffness (n = 5); superficial wound infections (n = 4); neurolysis (n = 1); hematoma (n = 3); deep wound infections (n = 2); percutaneous fistula (n = 1)
**Adjuvant ^90^yttrium RSO**					
Durr et al^23^ (n = 32 pts who underwent 37 surgeries) Pts included in Capellen et al^25^	Retrospective cohort (1996-2014) LOE: III	Diffuse PVNS F/U: 49^b^ mo	All pts had open Sv Adjuvant RSO administered after 26 surgeries (70%) No adjuvant RSO administered after 11 surgeries (30%)	Recurrence: all pts (24%; 9 surgeries); pts treated with RSO (23%; 6 surgeries); no RSO (27%; 3 surgeries)	NR
Gortzak et al ^24^ (n = 56)	Retrospective cohort (1991-2014) LOE: III	Diffuse TGCT F/U: 7.3^c^ yr	Pts had Sv + RSO (n = 34) or SV alone (n = 22) Types of Sv in RSO versus no RSO groups: open (29% versus 45%); arthroscopy (62% versus 40%); combined (3% versus 4%); unknown (6% versus 9%)—for comparisons, (P > 0.05)	Residual disease (asymptomatic recurrence): RSO (44%) versus no RSO (50%); (*P* > 0.05) No significant differences in overall physical health and mental health scores, perception of pain, and patient satisfaction	Sv + RSO group: none reported Sv alone group: infection (n = 1); skin ulcer from injection (n = 1) Degenerative changes at x-ray: RSO (n = 14; 41%); no RSO (n = 5; 23%)—none warranted treatment

CI = confidence interval, CRPS = chronic regional pain syndrome, DTGCT = diffuse-type giant cell tumor, DVT = deep vein thrombosis, Dx = diagnosis, F/U = follow-up, IKDC = International Knee Documentation Committee, KSS = Knee Society Score, LOE = level of evidence, MUA = manipulation under anesthesia; NR = not reported, OA = osteoarthritis, op = operative, open/arthroscopic = open posterior with arthroscopic anterior, open/open = open anterior with open posterior, postop = postoperatively, preop = preoperatively, pts = patients, PVNS = pigmented villonodular synovitis, RFS = recurrence-free survival, ROM = range of motion, RR = relative risk, RSO = radiosynoviorthesis, Sv = synovectomy, TGCT = tenosynovial giant cell tumor, TKA = total knee arthroplasty, Tx = treatment

a Percentage of patients, unless otherwise indicated.

b Median.

c Mean or average.

**Table 2:** Summary of Studies in Patients With TGCT in Various Sites

Study (No. of pts)	Study Design (Tx Period), LOE	Dx, F/U	Tx	Efficacy^a^	Complications^a^
**Surgery**					
Capellen et al^25^ (n = 105)	Retrospective cohort (1996-2014) LOE: III	PVNS in knee (n = 58), feet (n = 16), hand (n = 11), ankle (n = 9), hip (n = 4), elbow (n = 2), lower calf (n = 2), sacral joints (n = 1), upper calf (n = 1), shoulder (n = 1) Diffuse PVNS seen in 66 (54%) lesions F/U: 71^b^ mo (n = 103; 2 pts lost to F/U)	All pts had open Sv (n = 120 resections); some pts also had arthroscopy for diagnostic reasons	Recurrence all pts: 22 of 120 (18%) resections Recurrence at knee: 8 of 36 (22%) and 3 of 28 (11%) nodular lesion Persistent tumors at last f/u: 6 of 103 pts (5.8%) Recurrence in pts who underwent RSO: 7 of 27 pts (32%)	Postop: wound revisions due to hematomas (n = 2); necrosis of femoral condyle (n = 1); peroneal nerve palsy (n = 1); deep infection (n = 1); instability of collateral ligament at knee (n = 1)
Ma et al^26^ (n = 75)	Retrospective cohort (2000-2010) LOE: III	PVNS in knee (n = 52), hip (n = 18), ankle (n = 4), hand (n = 1) Localized PVNS (n = 8) or diffuse PVNS (n = 67) F/U: 40 mo^c^ (n = 60 pts kept all appointments)	All pts underwent open or arthroscopic Sv alone (n = 41 pts; n = 43 joints) or with arthroplasty (n = 34; n = 38 joints)	Recurrence on MRI: 17 joints with diffuse PVNS	No postop complications or infection
Mastboom et al^27^ (n = 941; n = 930 received tx at participating tertiary center; n = 823 with complete survival data)	Retrospective cohort (1990-2017) LOE: III	Localized TGCT in knee (n = 633), ankle (n = 119), foot (n = 58), hip (n = 37), hand (n = 33), wrist (n = 24), elbow (n = 14), shoulder (n = 9), other (n = 14) F/U: 37^b^ mo	Type of tx known for 930 pts: Open resection (n = 675), arthroscopic resection (n = 140), wait and see (n = 64), resection NS (n = 30), endoprosthetic reconstruction (n = 21)	Recurrence (n = 823): 12% LRFS for all pts: 3-yr, 88% (n = 388); 5-yr, 83% (n = 231); 10 yr, 79% (n = 66) RFS for tx naïve pts: 3-yr, 90% (n = 372); 5-yr, 86% (n = 223); 10-yr, 82% (n = 63) RFS: open (87%) versus arthroscopic surgery (80%) (*P* = 0.04); statistical significance lost in multivariate analysis Symptoms prior to treatment (n = 663-767) versus final F/U (n = 522-525): pain (73% versus 25%); swelling (66% versus 12%); joint stiffness (10% versus 4%); limited ROM (16% versus 5%)	Postop complications after surgery at tertiary center (n = 763): superficial wound infection (n = 11; 1%), deep wound infection (n = 1; 0.1%), joint stiffness (n = 5; 0.7%), hemorrhage (n = 1; 0.1%), neurovascular damage (n = 3; 0.4%), thrombosis (n = 3; 0.4%), other (n = 10; 1%)
Mastboom et al^11^ (n = 1192; n = 966 received surgery as primary tx and had complete data)	Retrospective cohort (1990-2017) LOE: III	Diffuse TGCT in knee (n = 758), hip (n = 124), ankle (n = 162), foot (n = 63), shoulder (n = 15), elbow (n = 17), wrist (n = 25), hand (n = 13), other (n = 15) F/U: 54^b^ mo	1-staged open Sv (n = 628), 2-staged open Sv (n = 187), arthroscopic Sv (n = 159), wait and see (n = 76), Sv not specified (n = 47)	First local recurrence (n = 966): 44% Total no. of recurrences (n = 425): 1 (63%); 2 (20%); ≥ 3 (17%) No evidence of disease at last F/U (n = 587): 66% LRFS for pts who received primary tx (n = 966): 3-yr, 62% (n = 474); 5-yr, 55% (n = 297); 10-yr, 40% (n = 89) LRFS for tx-naïve pts (n = 758): 3-yr, 70% (n = 372); 5-yr, 64% (n = 227); 10-yr, 50% (n = 70) Univariate analysis of 5-yr LRFS: Open (66%) versus arthroscopic surgery (54%) (*P* = 0.03); statistical significance lost in multivariate analysis Symptoms prior to treatment (n = 161–738) versus final F/U (n = 92–233): Pain (76% versus 37%); swelling (75% versus 24%); joint stiffness (21% versus 17%); limited ROM (28% versus 19%)	Postop complications after surgery at tertiary center (n = 906): superficial wound infection (n = 15; 2%), deep wound infection (n = 10; 1%), joint stiffness (n = 32; 4%), hemorrhage (n = 7; 1%), neurovascular damage (n = 15; 2%), thrombosis (n = 1; 0.1%), other (n = 25; 3%)
Palmerini et al^28^ (n = 294)^d^	Retrospective cohort (1998-2008) LOE: III	TGCT in knee (60%), ankle (16%), hip (11%), other sites (13%) Localized TGCT (n = 90) or diffuse TGCT (n = 196) F/U: 4.4^b^ yr	Open Sv (n = 171) or arthroscopic Sv (n = 66) None of the pts had EBRT or other local adjuvant tx Medical tx: Neoadjuvant imatinib (n = 2); disphosphonate (n = 1)	Local failure: All pts (28%); pts w/diffuse TGCT (36%); pts w/localized TGCT (14%) 5-yr LFFS: All pts (66%) 5-yr LFFS after arthroscopy versus open surgery: Localized TGCT (84% versus 72%; *P* = 0.4); diffuse TGCT (59% versus 61%; *P* = 0.8)	NR
Xie et al^29^ (n = 237)	Retrospective cohort (2005-2014) LOE: III	PVNS in knee (n = 175), hip (n = 43), ankle (n = 8), wrist (n = 6), shoulder (n = 2), elbow (n = 2), finger (n = 1) Localized or diffuse PVNS status: NR F/U: NR	Arthroscopic Sv (n = 129); open Sv (n = 108)	Recurrence: all pts (20%); knee (24%); hip (7%)	NR
**Drug therapies**					
Cassier et al^30^ (n = 29 enrolled; n = 28 evaluable for efficacy; n = 25 evaluable for safety; n = 17 in dose expansion cohort)	Phase 1 trial (2012-2014) LOE: II Primary objective: evaluate safety and tolerability, determine MTD or OBD	Locally advanced diffuse TGCT in knee (n = 15), foot or ankle (n = 8), hip (n = 4), wrist (n = 2) F/U: 12^b^ mo	Emactuzumab (1.5 h infusion every 2 wk) 3 + 3 dose-escalation phase followed by expansion phase	MTD not reached ORR: 86% CRs: 7% PRs: 79%	DLTs: None Most common AEs: facial edema (64%), asthenia (56%), pruritus (56%) SAEs (n = 5 pts): periorbital edema, lupus erythematosus, erythema, dermohypodermitis
Gelderblom et al^31^ (n = 56; n = 51 evaluable for efficacy)	Phase 2 trial (2010-2012) LOE: II Primary end point: proportion of pts progression-free at 12 wk	Inoperable progressive or relapsing PVNS or PVNS only resectable with mutilating surgery in knee (n = 29), ankle or foot (n = 13), hip or femoral neck (n = 7), hand or finger (n = 3), wrist (n = 2), ulna (n = 1), other (n = 1) F/U: 48 mo (n = 50)	Nilotinib (twice per day) 5 pts not evaluable for primary end point because discontinued study tx before week 12 Median duration of tx: 11 mo (IQR, 7.0-12.0)	% pts progression-free at 24 weeks: 90% Estimated mean proportion of pts progression-free at 24 weeks: 88% No CRs or PRs SD: 90% 31 pts (55%) completed 1 year of tx 1-yr PFS: 77%	% with AEs: ≥1 AE (98%); ≥1 TRAE (96%); AEs leading to treatment modification (41%); ≥1 grade 3 TRAE (11%) Grade 3 TRAE: headache, dizziness, hepatic disorders (n = 1); pruritus and toxidermia (n = 1); diarrhea (n = 1); increased γ-glutamyl transferase concentrations (n = 1); anorexia (n = 1); increased headache (n = 1)
Tap et al^32^ (n = 23 extension phase)	Phase 1 trial (2009-2014) LOE: II Primary objective: clinical benefit	TGCT in knee (n = 15), hip (n = 2), foot (n = 2), ankle (n = 2), elbow (n = 1), forearm (n = 1), metastatic (n = 1), with demonstrated progression within past 1 yr that was recurrent, inoperable, or resectable but requiring extensive surgery F/U: NR	Pexidartinib (once a day) Mean duration of tx: 254 d (range 15-585)	ORR: 52% (12 pts with PRs, 0 CRs) SD: 30% (7 pts) Responses usually occurred within 4 mo of tx start	Any TRAEs: 100% Most common: hair color changes (74%); fatigue (65%); nausea (39%); dysgeusia (26%); periorbital edema (26%); decreased appetite (26%); diarrhea (30%); vomiting (30%)
Tap et al^33^ (n = 120) Note: planned sample size of n = 126 for part 1^e^	Phase 3, double-blind, placebo-controlled, RCT (2015-2018) LOE: I Primary end point: ORR at week 25, based on blinded central MRI	Symptomatic advanced TGCT in knee (n = 73), ankle (n = 21), hip (n = 13), wrist (n = 4), foot (n = 3), shoulder (n = 2), spine (n = 2), elbow (n = 1), finger (n = 1), for whom surgery was not recommended F/U: 22 mo^b^	Part 1 (double-blind phase), twice daily pexidartinib (n = 61) or placebo (n = 59) for 24 wk Part 2, open-label pexidartinib at dose of pexidartinib or placebo ended on part 1 (n = 30)	ORR at week 25: Pexidartinib 39% (9 CRs, 15 PRs) versus placebo 0% (*P* < 0.0001) Difference (pexidartinib—placebo) in ROM change from baseline to week 25: mean + 8.9 ± 3.0 (*P* = 0.0043)	Pexidartinib versus placebo Most common any grade AEs: hsair color changes (67% versus 3%); fatigue (54% versus 36%); AST increase (39% versus 0%); nausea (38% versus 41%); ALT increase (28% versus 2%); diarrhea (20% versus 25%)
Verspoor et al^34^ (n = 62; n = 58 evaluable for efficacy)	Retrospective cohort (NR) LOE: III	Locally advanced, recurrent, or metastatic diffuse TGCT in knee (n = 35), ankle (n = 11), hip (n = 6), foot (n = 4), head and neck (n = 2), wrist (n = 2), shoulder (n = 1), elbow (n = 1) F/U: 52 mo^c^	Imatinib mesylate (once a day) Median duration of tx: 9 mo (IQR 5-26)	ORR: 31% CR: 4% PR: 27% SD: 65% Symptom improvement: 78% Median PFS: 18 mo (IQR 8-55) Median time to best response: 6 mo (range 1-23)	Most common AEs any grade: fatigue (50%); edema/fluid retention (48%); nausea (34%); skin rash/dermatitis (12%); other (26%) Grade 3-4 AEs: edema/fluid retention (2%); fatigue (2%); skin rash/dermatitis (3%); other (5%)

AE = adverse event, ALT = alanine aminotransferase, AST = aspartate aminotransferase, CI = confidence interval, CR = complete response, DLT = dose-limiting toxicity, DTGCT = diffuse-type giant cell tumor, Dx = diagnosis, EBRT = external beam radiation therapy, F/U = follow-up, IQR = interquartile range, KSS = Knee Society Score, LFFS = local failure-free survival, LOE = level of evidence, LRFS = local relapse-free survival; MTD = maximum tolerated dose, NR = not reported, NS = not specified, OA = osteoarthritis, OBD = optimal biologic dose, ORR = objective response rate, PFS = progression-free survival, postop = postoperatively, PR = partial response, pts = patients, PVNS = pigmented villonodular synovitis, RCT = randomized controlled trial, RFS = recurrence-free survival, ROM = range of motion, RSO = radiosynoviorthesis, SAE = serious adverse event, SD = stable disease, Sv = synovectomy, TGCT = tenosynovial giant cell tumor, TRAE = treatment-related adverse event, Tx = treatment

a Percentage of patients, unless otherwise indicated.

b Median.

c Mean or average.

d Study excluded patients with clinical history of > 1 local recurrence.

e Study protocol was amended to halt enrollment at 120 patients based on Data Monitoring Committee recommendation following cases of mixed or cholestatic hepatoxicity (2 patients in Tap et al, 2019 study, and other cases in pexidartinib's non-TGCT development plan).

**Table 3:** Summary of Studies in Patients With TGCT in Hand, Hip, Ankle, and Foot

Study (No. of pts)	Study Design (Tx period) LOE	Dx, F/U	Tx	Efficacy^a^	Complications^a^
**Hand**					
Koutserimpas et al^35^ (n = 36)	Retrospective cohort (2005-2015) LOE: III	GCTTS F/U: 21^b^ mo	Radical tumor resection	Recurrence: 11%	Postop: 11%
**Hip**					
Tibbo et al^36^(n = 25)	Retrospective cohort (1971-2013) LOE: III	Diffuse PVNS F/U: 10^b^ yr	THA	5-yr DFS: 100% 10-yr DFS: 100% 20-yr DFS: 100% Recurrence: 4%	Postop: 76% 5-yr SFR: 83% 10-yr SFR: 63%
**Foot and ankle**					
Korim et al^37^ (n = 30)	Retrospective cohort (2000-2010) LOE: III	DTGCT or localized PVNS F/U: 4^b^ yr	Open Sv	Recurrence: PVNS group (none); DTGCT group (12%)	NR

DFS = disease-free survival, DTGCT = diffuse-type giant cell tumor, Dx = diagnosis, F/U = follow-up, GCTTS = giant cell tumor of tendon sheath, LOE = level of evidence, NR = not reported, postop = postoperative, pts = patients, PVNS = pigmented villonodular synovitis, SFR = survivorship free from any revision, Sv = synovectomy, TGCT = tenosynovial giant cell tumor, THA = total hip arthroscopy, Tx = treatment

a Percentage of patients.

b Mean follow-up.

### Surgery

Only 11 of the 24 included studies reported comparative data regarding the treatment of patients with TGCT. Seven retrospective cohort studies compared open synovectomy with arthroscopic synovectomy.^11,14,16,20,21,27,28^ Note that some studies reported results in terms of recurrence rates (where lower percentages indicate better outcomes), and other studies reported results in terms of failure-free or relapse-free survival (where higher percentages indicate better outcomes).

Among the knee studies (Table 1), Colman et al^14^ noted a lower recurrence rate among patients treated with open posterior/arthroscopic anterior synovectomy compared with all-arthroscopic and open posterior/open anterior approaches (9% versus 62% versus 64%; *P* = 0.008). The results of this study need to be interpreted with caution because of potential selection bias in that patients with less anterior disease burden or intra-articular anterior disease (characteristics associated with a lower risk of recurrence) might have been preferentially treated with open posterior/arthroscopic anterior synovectomy rather than open/open approaches. Gu et al^16^ reported no significant difference in recurrence rate for open versus arthroscopic surgery (22% versus 6%; *P* ≥ 0.05) in patients with diffuse PVNS. A small knee study reported a lower rate of recurrence with initial open synovectomy than with arthroscopic synovectomy but did not include a statistical power analysis.^21^ A larger study by Patel et al^20^ found a significantly lower rate of recurrence with open versus arthroscopic synovectomy in patients with diffuse PVNS (44.8% versus 83.3%; *P* = 0.0004), and no significant difference was observed in patients with localized PVNS (8.6% versus 9.1%; *P* > 0.05) after a relatively short mean follow-up of 25 months.

Among the studies in patients with TGCT at heterogeneous sites (all predominantly knee), Palmerini et al^28^ reported no significant differences in 5-year local failure-free survival (LFFS) rates for open versus arthroscopic surgery in patients with localized TGCT (72% versus 84%; *P* = 0.4) and diffuse TGCT (61% versus 59%; *P* = 0.8) after a median follow-up of 4.4 years (Table 2). In a very large multicenter pooled cohort database study of patients with localized TGCT, Mastboom et al^27^ noted a higher rate of local relapse-free survival (LRFS) after open versus arthroscopic surgery (87% versus 80%; *P* = 0.04), but the statistical significance was lost in multivariate analysis. The same group reported similar results in patients with diffuse TGCT: univariate analysis yielded a 5-year relapse-free survival that favored open versus arthroscopic surgery (66% versus 54%; *P* = 0.03) and multivariate analysis eliminated the statistical significance.^11^

A retrospective cohort study reported no statistically significant differences in mean knee-function measures (Lysholm and Ogilvie-Harris scores) and lesion recurrence rate, with a higher incidence of chronic regional pain syndrome, but not of other complications, after arthroscopically assisted mini-open partial synovectomy versus arthroscopic excision of the lesion in 44 patients with localized PVNS (Table 1).^15^

### Radiosynoviorthesis

Both studies of adjuvant ^90^yttrium RSO^23,24^ reported similar rates of recurrence with and without RSO treatment in patients with diffuse disease in the knee (Table 1). One of these studies also found no significant differences in overall physical health and mental health scores, perception of pain, and patient satisfaction after a mean follow-up of 7.3 years.^24^

### Systemic Therapy

The five systemic drug therapy studies included 1 study of intravenous emactuzumab (an anti-CSF1R monoclonal antibody) in patients with locally advanced, diffuse TGCT^30^; 1 study of oral nilotinib (a CSF1R small molecule, tyrosine kinase inhibitor [TKI]) in patients with locally advanced PVNS^31^; two studies of oral pexidartinib (a CSF1R TKI) in patients with advanced TGCT^33^; and 1 study of oral imatinib mesylate (a CSF1R TKI) in patients with locally advanced, recurrent, or metastatic diffuse TGCT^34^ (Table 2).

In a phase 1 study, patients with locally advanced, diffuse TGCT achieved an 86% objective response rate (ORR), including 7% complete responses (CRs) and 79% partial responses (PRs), with emactuzumab biweekly infusions.^30^ The most commonly reported AEs were facial edema (64%), asthenia (56%), and pruritus (56%). A phase 2 study of twice-daily oral nilotinib yielded no CRs or PRs, but a 90% rate of stable disease (SD) among patients with inoperable progressive or relapsing PVNS, or PVNS only resectable with mutilating surgery.^31^ Many patients (41%) had AEs requiring treatment modification and 11% of patients had various grade 3 or higher AEs deemed to be related to nilotinib treatment.

Encouraging results of a phase 1 trial of the CSF1R inhibitor pexidartinib^32^ led to the ENLIVEN 24-week double-blind, placebo-controlled RCT of pexidartinib in patients with symptomatic advanced TGCT at various sites (primarily knee and ankle).^33^ Compared with the placebo group (n = 59), the twice-daily pexidartinib group (n = 61) had a higher ORR at week 25 (39% versus 0%; *P* < 0.0001) and a significant positive difference in mean range of motion change from baseline to week 25 (+8.9 ± 3.0; *P* = 0.0043). Hair color changes (67% versus 3%), fatigue (54% versus 36%), increased aspartate aminotransferase (39% versus 0%), and increased alanine aminotransferase (28% versus 2%) were the most common AEs reported at higher rates in the pexidartinib versus placebo group, respectively. More patients in the pexidartinib versus the placebo group had serious AEs (13% versus 2%), which included three patients (5%) with aspartate aminotransferase and alanine aminotransferase ≥ 3 × upper limit of normal, along with total bilirubin and alkaline phosphatase ≥ 2 × upper limit of normal. The only death reported in the study (in a patient with a history of cardiovascular disease) was unrelated to pexidartinib treatment. The cases of mixed or cholestatic hepatotoxicity seen in the pexidartinib arm and other cases reported in non-TGCT studies led to early closing of study recruitment after 120 (95%) of the planned 126 patients had enrolled and led to stoppage of crossover from placebo to the pexidartinib arm in the nonrandomized extension phase of the trial.

A retrospective cohort study (n = 62) reported a 31% ORR, including 4% CR and 27% PR, and 65% SD with once-daily oral imatinib in patients with advanced or recurrent diffuse TGCT.^34^ This study included long-term follow-up of 29 patients previously reported in a 2012 publication^38^ plus 33 additional consecutive patients treated since that report. The most commonly reported AEs were fatigue (50%), edema/fluid retention (48%), and nausea (34%). Very few patients experienced grade 3 to 4 AEs.

## Discussion

Surgical resection continues to be the mainstay of treatment for patients with localized and diffuse TGCT. As reiterated in the results of this systematic review, to manage progressive disease or disease recurrences, many patients face the daunting prospect of undergoing multiple surgeries. Although several studies have tried to answer the question of whether open or arthroscopic resection leads to better outcomes in patients with TGCT, none have compared the outcomes with systemic treatment versus surgery.

In our analysis, among the studies that compared open synovectomy with arthroscopic synovectomy in patients with diffuse disease, three studies reported no statistically significant differences in recurrence rate (64% versus 62%^14^; 22% versus 6%)^16^ or 5-year LRFS (61% versus 59%)^28^ and three studies reported that open surgery was associated with lower recurrence (29% versus 94%^21^; 44.8% versus 83.3%)^20^ or higher 5-year LRFS (66% versus 54%).^11^ Among the three studies that compared open versus arthroscopic synovectomy in patients with localized disease, two studies found no statistically significant differences in recurrence rate (8.6% versus 9.1%)^20^ or 5-year LRFS (72% versus 84%)^28^ and 1 study found that open surgery was associated with higher LRFS (87% versus 80%).^27^ The two largest TGCT cohort studies^11,27^ included in our review (and not included in previous systematic reviews) noted that the statistically significant improvements in LRFS associated with open versus arthroscopic surgery observed by univariate analyses disappeared following multivariate analyses.

Although one might assume that complications of open surgery would be higher than complications of arthroscopic surgery, there are no data from head-to-head RCTs to support or refute this supposition. Existing published data are circumstantial and sparse. Thus far, we have not found clear differences in complication rates between open or arthroscopic one- or two-staged surgery.

Thus, regarding surgical treatments, the results of our systematic review align with previous reports of somewhat contradictory results and high risk of disease progression, especially in patients with diffuse TGCT. In a 2017 systematic review of long-term clinical outcomes and rates of recurrence for open or arthroscopic excision in patients with GCTTS involving four types of joints (ie, shoulder, hip, knee, and ankle), Gouin's group concluded that although arthroscopy showed effectiveness for localized disease for all four joints, the data supported arthroscopy only for the knee joint because of the risk of osteoarthritis degradation.^39^ Unfortunately, the findings from our study do not bring more clarity to the open versus arthroscopic debate. At present, more and more centers use a hybrid arthroscopic anterior and open posterior approach in patients with diffuse TGCT of the knee, including two studies^14,20^ that met the inclusion criteria for our analysis and 1 study that had too few patients.^40^

Interpretation of the combined surgical studies is restricted by study design limitations, particularly the retrospective design and comparisons of noncontemporaneous cohorts (with study periods spanning more than a decade in most cases). Lack of control for potential sources of bias such as differences in surgeon's experience and learning curve, patient clinical characteristics, outcome measures, and length of follow-up are also limiting factors. Better quality studies—especially RCTs or case-matched analyses—are needed to determine which surgical techniques are optimal for TGCT. RCTs are expensive and difficult to perform in a surgical setting, especially for a rare disease such as TGCT.

Among the four agents evaluated in clinical trials identified in our literature review, emactuzumab,^30^ imatinib,^34^ and pexidartinib^32,33^ have shown promising efficacy in ORR, demonstrating measurable reductions in tumor burden. The clinical development of emactuzumab in TGCT remains unclear after promising phase 1 results published in 2015^30^; since then, no new data have been reported and there are currently no ongoing studies. Since its initial US approval in 2001, imatinib had gained several indications for various hematologic malignancies, dermatofibrosarcoma protuberans, and gastrointestinal stromal tumors^41^; its administration to patients with TGCT would be off-label. The ENLIVEN study met its primary end point with a higher ORR for pexidartinib compared with the placebo.^33^ These results supported the August 2019 United States FDA approval of pexidartinib for the treatment of adults with symptomatic TGCT associated with severe morbidity or functional limitations and not amenable to improvement with surgery. Because the drug was approved with a boxed warning regarding the risk of serious and potentially fatal liver injury, it is prescribed and dispensed solely via a manufacturer-supported Risk Evaluation and Mitigation Strategy safety program.^42^ As of August 2019, the National Comprehensive Cancer Network clinical practice guidelines for soft tissue sarcoma list only two systemic therapy agents for TGCT: pexidartinib as a category 1 recommendation and imatinib as a category 2A recommendation.^10^ In the context of pexidartinib, additional research and clinical experience are warranted to better understand optimal patient selection, treatment course, patient adherence to treatment, and prevention and management of toxicities.

Our systematic review was limited by the restriction of PubMed and Ovid searches to studies published from 2013 to the present to reflect the most current treatment practices for patients with TGCT. Other limitations included exclusion of unpublished studies, studies of EBRT as the primary treatment modality, and studies with fewer than 20 patients. By limiting studies to those with histologically confirmed patients, we were able to more accurately reflect the current standard of care.

Investigation of other systemic therapies for TGCT is currently limited to a few ongoing studies. These include an open-label phase 1 study (NCT03069469) of the oral CSF1R inhibitor DCC-3014 in patients with solid tumors, including tumor types with high colony-stimulating factor 1 expression such as TGCT and an ongoing Japanese randomized placebo-controlled double-blind phase 2 trial that is evaluating the nonsteroidal anti-inflammatory drug zaltoprofen in patients with diffuse TGCT and unresectable localized TGCT.^43^

One important question that needs to be addressed is what is the best outcome measure to assess treatment in patients with TGCT. The studies of surgical treatments assessed local progression or local recurrence (which is often not a recurrence but a local progression). Would different measures be more appropriate for different treatment modalities (eg, surgery versus systemic therapy)? Because cure is rare, should we be assessing SD without complaints?

In 2012, van der Heijden et al^4^ suggested that a multidisciplinary approach is required to improve outcomes of patients with recurrent and refractory diffuse disease. This approach should include dedicated magnetic resonance imaging, histologic assessment, and planned surgery with adjuvant radiotherapy or systemic targeted therapy. The subsequent collective experience supports the notion that multidisciplinary treatment is key to better outcomes for patients with TGCT, particularly given the tumor's inflammatory response and ensuing symptoms. The US FDA approval of pexidartinib, the first systemic drug therapy for selected adults with symptomatic TGCT, augments the armamentarium available for multidisciplinary treatment. Questions remain regarding the optimal combination and sequencing of available therapies. Surgeons, medical oncologists, and other clinicians have a unique opportunity to work together to improve health and quality of life outcomes for this patient population.

## Conclusions

The significant rates of recurrence and risks associated with surgery point to the need for novel systemic treatments for patients with symptomatic advanced TGCT. The recent approval of a systemic therapy for selected adults with symptomatic TGCT underscores the need for improved and more coordinated multidisciplinary care.
